# Validation of the Kazakh version of the movement disorder Society-Unified Parkinson's disease rating scale

**DOI:** 10.1016/j.prdoa.2024.100232

**Published:** 2024-01-06

**Authors:** Saltanat Abdraimova, Zhanybek Myrzayev, Altynay Karimova, Altynay Talgatkyzy, Talgat Khaibullin, Gulnaz Kaishibayeva, Sandugash Elubaeva, Karlygash Esembekova, Dongrak Choi, Pablo Martinez-Martin, Christopher G. Goetz, Glenn T. Stebbins, Sheng Luo, Chingiz Shashkin, Nazira Zharkinbekova, Rauan Kaiyrzhanov

**Affiliations:** a South Kazakhstan Medical Academy, Department of Neurology, Psychiatry, Rehabilitology and Neurosurgery, Shymkent, Kazakhstan; b International Research Institute of Postgraduate Education, Department of Neurosurgery and Neurology, Almaty, Kazakhstan; c Kazakh National University n.a. Al-Farabi, Department of Biomedicine, Biophysics and Neuroscience, Almaty, Kazakhstan; d “Semey Medical University”, Department of Neurology, Ophthalmology, and Otorhinolaryngology, Semey, Kazakhstan; e Institute of Neurology and Neurorehabilitation n.a. Smagul Kaishibayev, Almaty, Kazakhstan; f Nazarbayev University, Graduate School of Education (NU GSE), Educational Leadership (School Education), Astana, Kazakhstan; g Nazarbayev Intellectual School of Physics and Mathematics, Semey, Kazakhstan; h Department of Biostatistics and Bioinformatics, Duke University, Durham, NC, USA; i Center for Networked Biomedical Research in Neurodegenerative Diseases (CIBERNED), Carlos III Institute of Health, Madrid, Spain; j Department of Neurological Sciences, Rush University Medical Center, Chicago, IL, USA; k Institute of Neurology, University College London, Department of Neuromuscular Disorders, Queen Square, London, UK

**Keywords:** MDS-UPDRS, Parkinson’s Disease, Rating scale, Validation

## Abstract

**Background and Purpose:**

The International Movement Disorder Society revision of the Unified Parkinson’s Disease Rating Scale (MDS-UPDRS) is widely used in the assessment of the severity of Parkinson’s disease (PD). This study aimed to validate the Kazakh version of the MDS-UPDRS, explore its dimensionality, and compare it to the original English version.

**Methods:**

The validation was conducted in three phases: first, the English version of the MDS-UPDRS was translated into Kazakh and thereafter back-translated into English by two independent teams; second, the Kazakh version underwent a cognitive pretesting; third, the Kazakh version was tested in 360 native Kazakh-speaking PD patients. Both confirmatory and exploratory factor analyses were performed to validate the scale. We calculated the comparative fit index (CFI) for confirmatory factor analysis and used unweighted least squares for exploratory factor analysis.

**Results:**

The CFI was higher than 0.90 for all parts of the scale, thereby meeting the pre-set threshold for the official designation of a validated translation. Exploratory factor analysis also showed that the Kazakh MDS-UPDRS has the analogous factors structure in each part as the English version.

**Conclusions:**

The Kazakh MDS-UPDRS had a consistent overall structure as the English MDS-UPDRS, and it was designated as the official Kazakh MDS-UPDRS, which can reliably be used in the Kazakh-speaking populations. Presently, Kazakhstan stands as the sole country in both Central Asia and Transcaucasia with an MDS-approved translated version of the MDS-UPDRS. We expect that other Central Asian and Transcaucasian countries will embark on the MDS Translation Program for MDS-UPDRS in the near future.

## 1 Introduction

Parkinson's disease (PD) is a common neurodegenerative disease characterized by a heterogeneous clinical presentation [1]. The disease predominantly affects the elderly population, but cases also occur before the age of 50 years [2]. The pathobiology of the disease includes the selective loss of dopaminergic neurons in the substantia nigra pars compacta and the involvement of other structures of the central nervous system and peripheral tissues [3]. As a consequence of these factors, there are clinical motor and non-motor manifestations of the disease [4].

The Unified Parkinson’s Disease Rating Scale (UPDRS) scale, developed in 1980, has been widely used to assess the stages and severity of PD [5]. However, this scale had several limitations as identified by the Task Force for Rating Scales in PD [6]. These limitations included ambiguity in terminology formulation, the absence of a standardized survey structure for raters, certain metric defects, and the exclusion of numerous significant non-motor symptoms. To address these limitations, the Movement Disorders Society (MDS) revised the UPDRS in 2008 [7]. The revised version, currently known as the MDS-UPDRS, underwent rigorous clinimetric testing, emerging as a robust and effective tool for both scientific research and daily clinical practice. The MDS-UPDRS not only overcame the limitations of its predecessor but also retained the strengths of the UPDRS [8].

After the MDS-UPDRS was formally introduced, MDS developed a program to translate the English version of the MDS-UPDRS into other languages, validate, and approve it. For this purpose, MDS has established a rigorous protocol and criteria for conducting the study. Several official translations are now available on the MDS website [9], [10], [11], [12], [13], [14], [15], [16], [17]. We set out to translate the MDS-UPDRS scale into the Kazakh language, validate this translation, and compare it with the original English version.

Kazakhstan is the largest country in Central Asia, located between Europe and East Asia [18]. The Kazakh language is part of the Turkic language group, spoken by around 200 million people worldwide across Eastern Europe, the Middle East, Central Asia, East Asia, and Siberia [19]. The Turkic language family encompasses over 35 documented languages [20]. The linguistic features almost universal within the Turkic language family are vowel harmony, agglutination, subject-object-verb order, and lack of grammatical gender [21]. Currently, the MDS UPDRS is officially translated only into Turkish, a representative of the Turkic languages. However, there's a noteworthy linguistic diversity within the Turkic group. Among them, the Kazakh language holds prominence in the largest Central Asian country, Kazakhstan. Expanding the accessibility of the MDS UPDRS to individuals with PD, who belong to Turkic-speaking communities, could be achieved by translating it into Kazakh. This step would significantly broaden the scope of the scale’s impact to a more extensive range of patients across the diverse Turkic-speaking populations.

In this article, we report the translation of the scale and the results of the clinometric testing of the Kazakh version of the MDS-UPDRS scale.

## 2 Materials and methods

This was an observational cross-sectional multicenter study. The study involved 360 Kazakh-speaking patients with a confirmed diagnosis of PD according to the new 2015 MDS diagnostic criteria [22] and at various stages of the disease according to Hoehn & Yahr [23].

The forward translation phase of the MDS-UPDRS was performed by a team of neurologists and movement disorder specialists, fluent in the Kazakh and English languages, and professional translators. Then there was a back-translation phase, from the Kazakh to the English language, by another group of movement disorders specialists, also fluent in the Kazakh and English languages and professional translators, who did not participate in the original translation. The back-translation was reviewed by the administrative team responsible for the overall translation program (Glenn Stebbins, Sheng Luo, Pablo Martinez-Martin).

### 2.1 Cognitive pretesting

A Cognitive Pretesting was conducted to assess the intelligibility of the questions and instructions, and to collect feedback in terms of the difficulty of the task, as well as interest, attention span, presence or absence of discomfort [12].

All items on the MDS-UPDRS questionnaire were pretested. After the pre-test cognitive testing, additional adjustments were made to the forward and back-translations if required. After improving the quality of the translations and after taking into account the results of the cognitive pretesting, the final Kazakh translation was obtained. Overall, 5 movement disorder specialists participated in the pre-testing study, and 15 PD patients were involved.

### 2.2 Validation of the Kazakh version of the MDS-UPDRS

A team of experienced Kazakh movement disorder specialists from 4 centers participated in the validation phase. All raters were members of the MDS and were trained to use the MDS-UPDRS as part of the MDS program [24]. The study was approved by the Institutional Review Board (IRB) of the South Kazakhstan Medical Academy 044-65/08-(48) 2021/03/16. Patients who signed informed consent participated in the study. The personal data of the patients were deidentified by assigning study codes. The deidentified data was securely sent to MDS for statistical analysis.

### 2.3 Data analysis

#### 2.3.1 Factor analysis

M−plus, Version 7.4 was used to perform the primary confirmatory and secondary exploratory factor analyses as the variables are categorical. We used an adjusted weighted least square (WLSMV) approach to factor estimation that minimizes the weighted sum of squared differences between observed and estimated correlation matrices not counting diagonal elements. To assist in the interpretation of the factors we used an orthogonal CF-VARIMAX rotation that constrains the factors to be uncorrelated. For comparison, we used the Chi-square statistic and its degree of freedom, 90 % CI of RMSEA. To evaluate the presence of local response dependence modification indices (MI) was used.

The sample size for the translation study was based on the need for at least 5 subjects per item of the scale to perform the statistical analysis [25], [26]. The MDS-UPDRS has 65 items and a sample of 350 patients is considered acceptable for validation of translated versions. Any participant with missing values within a Part was removed from the analysis of that Part only. Thus, the sample size from Part to Part could vary.

#### 2.3.2 Primary analysis

For the primary analysis of the Kazakh data, we conducted a confirmatory factor analysis (CFA) to determine if the factor structure for the English language MDS-UPDRS [6], could be confirmed in data collected using the Kazakh translation. This was the primary question of interest. The CFA was conducted separately for MDS-UPDRS Parts I to IV with the Kazakh data constrained to fall into the factors defined in the English language data. We evaluated the CFA results based on the Comparative Fit Index (CFI). According to the protocol, to establish a successful translation and to designate that translation as an OFFICIAL MDS translation of the MDS-UPDRS, we required that the CFI for each Part (I-IV) of the translated MDS-UPDRS be 0.90 or greater relative to the English language version. The mean and variance adjusted weighted least square (WLSMV) estimator is used to confirm model fit.

#### 2.3.3 Secondary analysis

As a secondary analysis, we conducted an exploratory factor analysis for the Kazakh version of the MDS-UPDRS Parts I-IV to explore the underlying factor structure without the constraint of a pre-specified factor structure. Once the factors are chosen, an item was retained in a factor if the factor loading for that item was 0.40 or greater. To assist interpretation of the factors, an orthogonal CF-VARIMAX rotation was used which sets the factors to be uncorrelated.

## 3 Results

### 3.1 Cognitive pretesting

Fifteen patients with PD and their examiners were interviewed using the type of structured interview format typical for cognitive pretesting. No problems were identified for the raters. Two of the 15 patients interviewed had difficulty understanding the term “fatigue”. No other patient-identified difficulties were noted. Slight modifications of the scale were recommended from this round of testing. The modified version of the scale was approved by the MDS as the Official Working Document of the Kazakh MDS-UPDRS and was administered to a larger group of PD patients for further testing.

### 3.2 Study population characteristics

The demographic characteristics of the sample are shown in Table 1. The Kazakh dataset included 360 native Kazakh-speaking patients with PD (mean age 62.6 ± 8.8 years, 40 % males), with a mean disease duration of 5.6 ± 4.0 years, who were examined using the MDS-UPDRS. All individuals demonstrated fluency in Kazakh. The majority (45 %) possessed a secondary education, while 28 % held a university degree. Only 27 % of patients had an educational attainment below the secondary level. The entire spectrum of disease severity was encompassed in the study. According to the Hoehn & Yahr classification, 93 individuals (25.8 %) were classified in stage 1, 87 (24.2 %) in stage 2, 129 (35.8 %) in stage 3, 40 (11.1 %) in stage 4, and 11 (3.1 %) in stage 5.

**Table 1:** The demographic characteristics of the patients.

	Total	Male n (%)	Age	Disease duration
Kazakh	360	144 (40)	62.6 ± 8.8	5.6 ± 4.0
English Reference Standard	876	554 (63.2)	67.5 ± 10.9	8.3 ± 6.7

Hoehn and Yahr Stages (%)						
	N	1	2	3	4	5
Kazakh	360	93 (25.8)	87 (24.2)	129 (35.8)	40 (11.1)	11 (3.1)
English	876	63 (7.3)	467 (53.9)	174 (20.1)	109 (12.6)	53 (6.1)

Table 1 in the Supplementary Material presents the distributions of the item responses provided by the Kazakh-speaking and English-speaking groups.

### 3.3 Primary analysis

#### 3.3.1 Confirmatory factor analyses (CFA)

Table 2 displays the Comparison of Kazakh and English Confirmatory Factor Analysis (CFA) results by MDS-UPDRS Parts. All four parts of the Kazakh MDS-UPDRS satisfied the pre-specified criterion of CFI ≥ 0.90 in comparison with the English-language factor structure.

**Table 2:** Comparison of Kazakh and English Confirmatory Factor Analysis (CFA) results by MDS-UPDRS Parts.

MDS-Parts		Kazakh	English
Part I (13 items)	CFI	0.971	0.955
	RMSEA (90 % CI)	0.068 (0.055, 0.080)	0.052 (0.045, 0.060)
	Chi-square	169.61	211.87
	Degree of freedom	64	64
	Patients N	360	846
Part II (13 items)	CFI	0.975	0.974
	RMSEA (90 % CI)	0.130 (0.118, 0.141)	0.085 (0.077, 0.092)
	Chi-square	436.75	440.49
	Degree of freedom	62	62
	Patients N	360	851
Part III (33 items)	CFI	0.903	0.949
	RMSEA (90 % CI)	0.160 (0.156, 0.164)	0.068 (0.065, 0.070)
	Chi-square	4828.39	2207.27
	Degree of freedom	474	474
	Patients N	360	801
Part IV (6 items)	CFI	0.999	0.999
	RMSEA (90 % CI)	0.055 (0.015, 0.092)	0.037 (0.013, 0.061)
	Chi-square	16.69	17.50
	Degree of freedom	8	8
	Patients N	360	848

RMSEA- the root mean square error of approximation

Table 3 presents the Part III items with minimum modification indices (MI) > 50. The findings suggest a minimal impact of local response dependence on the CFI value.

**Table 3:** Part III Modification Indices (MI) with minimum MI > 50.

Item 1	Item 2	MI
Rest tremor amplitude (RUE)	Postural tremor (R)	174.51
Rigidity (LLE)	Rigidity (LUE)	80.14
Kinetic tremor (R)	Postural tremor (R)	65.37
Leg agility (L)	Toe tapping (L)	60.97
Rest tremor amplitude (RUE)	Pronation-supination movements (L)	56.81
Rigidity (RLE)	Rigidity (RUE)	56.56
Rest Tremor amplitude (LUE)	Rest tremor amplitude (RUE)	53.89
Rest Tremor amplitude (LUE)	Postural Tremor (R)	53.16
Rest Tremor amplitude (RUE)	Hand Movements (L)	52.59
Constancy of rest	Rest Tremor amplitude (RUE)	52.00
Toe Tapping (L)	Pronation-supination movements (R)	50.33

### 3.4 Secondary analysis

#### 3.4.1 Exploratory factor analyses (EFA)

The factor structure of the EFA for the English version has been used as the basis for all CFAs, but our EFA of the Kazakh data set differs from that of the English-language data set in some aspects. The results of the EFA for the English and Kazakh versions are shown in Supplementary Table 2, including the number of factors and their associated eigenvalues and percent variance in Supplementary Figure 1–4.

## 4 Discussion

This study validates the translated Kazakh version of the MDS-UPDRS, demonstrating consistency with the original English version and meeting specified criteria for factor analyses.

Rating scales are used for patient assessment, follow-up, and for making decisions in clinical practice as well as for research, especially clinical trials. The implementation of these scales internationally requires the validation of individual language editions to guarantee their uniformity of application, usefulness, and credibility [27]. Independent validation of health measures is necessary to confirm or reject the findings obtained by the developers of the instrument. Reliability, validity, and responsiveness are key properties of a scale: they are indicative of its quality as a measurement instrument and they have to be carefully tested [28], [29].

The MDS-UPDRS Kazakh version is henceforth designated as an official MDS scale version of the MDS-UPDRS and is available from the MDS website (https://www.movementdisorders.org/MDS-Files1/Education/Rating-Scales/MDS-UPDRS_Kazakh_Official_Translation_FINAL.pdf). Currently, Kazakhstan is the only country in Central Asia and Transcaucasia that has the MDS approved version of the MDS-UPDRS translated to the regional language. We expect that other Central Asian and Transcaucasian countries, speaking the languages of Turkic origin, will embark on the MDS Translation Program for MDS-UPDRS in the near future. The availability of the Kazakh version of the MDS UPDRS translation will be an invaluable point of reference for these countries, especially considering the unique linguistic traits of Turkic languages.

The availability of the Kazakh version of the MDS-UPDRS holds an immediate practical advantage, enabling Kazakhstan’s qualification for participation in international clinical trials utilizing the MDS-UPDRS. This not only broadens access for Kazakh patients to novel clinical treatments but also creates opportunities for Kazakh medical professionals to contribute to global research. Looking ahead, with the patient population and research team now in place, we intend to conduct a one-year follow-up with these 360 patients, analyzing and comparing scores across the four parts of the MDS-UPDRS to derive a numerical measure of disease progression within this population.

## Declaration of competing interest

The authors declare that they have no known competing financial interests or personal relationships that could have appeared to influence the work reported in this paper.
