# Supplementary material for: Validation of the Kazakh version of the movement disorder Society-Unified Parkinson's disease rating scale
Source: Clin Park Relat Disord. 2024 Jan 6;10:100232. doi: 10.1016/j.prdoa.2024.100232 (PMC10826294; doi:10.1016/j.prdoa.2024.100232)
Supplement: Supplementary data 1 [file mmc1.docx]

**Supplementary**

**Table 1**

*Distribution of MDS-UPDRS Responses by Language*

*Part 1*

|  | Kazakh | | English | |  | Kazakh | | English | |
| --- | --- | --- | --- | --- | --- | --- | --- | --- | --- |
| Cognitive  impairment | **Freq** | **Percent** | **Freq** | **Percent** | **Hallucinations**  **and**  **psychosis** | **Freq** | **Percent** | **Freq** | **Percent** |
| 0 | 96 | 26.67 | 428 | 48.86 | 0 | 211 | 58.61 | 687 | 78.42 |
| 1 | 120 | 33.33 | 256 | 29.22 | 1 | 86 | 23.89 | 89 | 10.16 |
| 2 | 112 | 31.11 | 121 | 13.81 | 2 | 54 | 15 | 51 | 5.82 |
| 3 | 27 | 7.5 | 53 | 6.05 | 3 | 9 | 2.5 | 35 | 4 |
| 4 | 5 | 1.39 | 17 | 1.94 | 4 | 0 | 0 | 13 | 1.48 |
| Total | 360 | 100 | 875 | 99.89 | Total | 360 | 100 | 875 | 99.89 |
| Depressed  mood | **Freq** | **Percent** | **Freq** | **Percent** | **Anxious**  **mood** | **Freq** | **Percent** | **Freq** | **Percent** |
| 0 | 167 | 46.39 | 471 | 53.77 | 0 | 84 | 23.33 | 413 | 47.15 |
| 1 | 107 | 29.72 | 265 | 30.25 | 1 | 143 | 39.72 | 307 | 35.05 |
| 2 | 54 | 15 | 81 | 9.25 | 2 | 112 | 31.11 | 96 | 10.96 |
| 3 | 28 | 7.78 | 45 | 5.14 | 3 | 16 | 4.44 | 41 | 4.68 |
| 4 | 4 | 1.11 | 12 | 1.37 | 4 | 5 | 1.39 | 17 | 1.94 |
| Total | 360 | 100 | 874 | 99.77 | Total | 360 | 100 | 874 | 99.77 |
| Apathy | **Freq** | **Percent** | **Freq** | **Percent** | **Features of**  **DDS** | **Freq** | **Percent** | **Freq** | **Percent** |
| 0 | 103 | 28.61 | 584 | 66.67 | 0 | 235 | 65.28 | 747 | 85.27 |
| 1 | 137 | 38.06 | 141 | 16.1 | 1 | 84 | 23.33 | 57 | 6.51 |
| 2 | 92 | 25.56 | 88 | 10.05 | 2 | 28 | 7.78 | 44 | 5.02 |
| 3 | 23 | 6.39 | 52 | 5.94 | 3 | 10 | 2.78 | 19 | 2.17 |
| 4 | 5 | 1.39 | 8 | 0.91 | 4 | 3 | 0.83 | 6 | 0.68 |
| Total | 360 | 100 | 873 | 99.66 | Total | 360 | 100 | 873 | 99.66 |
| Sleep  problems | **Freq** | **Percent** | **Freq** | **Percent** | **Daytime**  **sleepiness** | **Freq** | **Percent** | **Freq** | **Percent** |
| 0 | 47 | 13.06 | 280 | 31.96 | 0 | 100 | 27.78 | 212 | 24.2 |
| 1 | 97 | 26.94 | 202 | 23.06 | 1 | 130 | 36.11 | 216 | 24.66 |
| 2 | 123 | 34.17 | 207 | 23.63 | 2 | 109 | 30.28 | 364 | 41.55 |
| 3 | 77 | 21.39 | 140 | 15.98 | 3 | 20 | 5.56 | 59 | 6.74 |
| 4 | 16 | 4.44 | 40 | 4.57 | 4 | 1 | 0.28 | 16 | 1.83 |
| Total | 360 | 100 | 869 | 99.2 | Total | 360 | 100 | 867 | 98.97 |
| Pain and other sensations | **Freq** | **Percent** | **Freq** | **Percent** | **Urinary problems** | **Freq** | **Percent** | **Freq** | **Percent** |
| 0 | 81 | 22.5 | 303 | 34.59 | 0 | 120 | 33.33 | 325 | 37.1 |
| 1 | 117 | 32.5 | 289 | 32.99 | 1 | 112 | 31.11 | 281 | 32.08 |
| 2 | 120 | 33.33 | 130 | 14.84 | 2 | 84 | 23.33 | 137 | 15.64 |
| 3 | 37 | 10.28 | 106 | 12.1 | 3 | 37 | 10.28 | 88 | 10.05 |
| 4 | 5 | 1.39 | 39 | 4.45 | 4 | 7 | 1.94 | 38 | 4.34 |
| Total | 360 | 100 | 867 | 98.97 | Total | 360 | 100 | 869 | 99.2 |
| Constipation  problems | **Freq** | **Percent** | **Freq** | **Percent** | **Lightheaded**  **ness** | **Freq** | **Percent** | **Freq** | **Percent** |
| 0 | 49 | 13.61 | 384 | 43.84 | 0 | 108 | 30 | 490 | 55.94 |
| 1 | 110 | 30.96 | 287 | 32.76 | 1 | 151 | 41.94 | 216 | 24.66 |
| 2 | 142 | 39.44 | 119 | 13.58 | 2 | 79 | 21.94 | 103 | 11.76 |
| 3 | 53 | 14.72 | 70 | 7.99 | 3 | 20 | 5.56 | 51 | 5.82 |
| 4 | 6 | 1.67 | 9 | 1.03 | 4 | 2 | 0.56 | 9 | 1.03 |
| Total | 360 | 100 | 869 | 99.2 | Total | 360 | 100 | 869 | 99.2 |
| Fatigue | **Freq** | **Percent** | **Freq** | **Percent** |  | | | | |
| 0 | 42 | 11.67 | 217 | 24.77 |  |  |  |  |  |
| 1 | 132 | 36.67 | 335 | 38.24 |  |  |  |  |  |
| 2 | 141 | 39.17 | 184 | 21 |  |  |  |  |  |
| 3 | 37 | 10.28 | 81 | 9.25 |  |  |  |  |  |
| 4 | 8 | 2.22 | 50 | 5.71 |  |  |  |  |  |
| Total | 360 | 100 | 867 | 98.97 |  |  |  |  |  |

*Part 2*

|  | Kazakh | | English | |  | Kazakh | | English | |
| --- | --- | --- | --- | --- | --- | --- | --- | --- | --- |
| Speech | **Freq** | **Percent** | **Freq** | **Percent** | **Saliva and drooling** | **Freq** | **Percent** | **Freq** | **Percent** |
| 0 | 188 | 52.22 | 252 | 28.77 | 0 | 122 | 33.89 | 341 | 38.93 |
| 1 | 87 | 24.17 | 236 | 26.94 | 1 | 107 | 29.72 | 115 | 13.13 |
| 2 | 55 | 15.28 | 233 | 26.6 | 2 | 84 | 23.33 | 203 | 23.17 |
| 3 | 21 | 5.83 | 126 | 14.38 | 3 | 41 | 11.39 | 157 | 17.92 |
| 4 | 9 | 2.5 | 22 | 2.51 | 4 | 6 | 1.67 | 53 | 6.05 |
| Total | 360 | 100 | 869 | 99.2 | Total | 360 | 100 | 869 | 99.2 |
| Chewing and swallowing | **Freq** | **Percent** | **Freq** | **Percent** | **Eating tasks** | **Freq** | **Percent** | **Freq** | **Percent** |
| 0 | 197 | 54.72 | 549 | 62.67 | 0 | 107 | 29.72 | 363 | 41.44 |
| 1 | 99 | 27.5 | 230 | 26.26 | 1 | 133 | 36.94 | 265 | 30.25 |
| 2 | 45 | 12.5 | 54 | 6.16 | 2 | 82 | 22.78 | 187 | 21.35 |
| 3 | 17 | 4.72 | 34 | 3.88 | 3 | 34 | 9.44 | 42 | 4.79 |
| 4 | 2 | 0.56 | 3 | 0.34 | 4 | 4 | 1.11 | 10 | 1.14 |
| Total | 360 | 100 | 870 | 99.32 | Total | 360 | 100 | 867 | 98.97 |
| Dressing | **Freq** | **Percent** | **Freq** | **Percent** | **Hygiene** | **Freq** | **Percent** | **Freq** | **Percent** |
| 0 | 70 | 19.44 | 220 | 25.11 | 0 | 73 | 20.28 | 342 | 39.04 |
| 1 | 154 | 42.78 | 322 | 36.76 | 1 | 150 | 41.67 | 367 | 41.89 |
| 2 | 79 | 21.94 | 211 | 24.09 | 2 | 70 | 19.44 | 88 | 10.05 |
| 3 | 43 | 11.94 | 76 | 8.68 | 3 | 49 | 13.61 | 33 | 3.77 |
| 4 | 14 | 3.89 | 42 | 4.79 | 4 | 18 | 5 | 38 | 4.34 |
| Total | 360 | 100 | 871 | 99.43 | Total | 360 | 100 | 868 | 99.09 |
| Handwriting | **Freq** | **Percent** | **Freq** | **Percent** | **Doing hobbies and other activities** | **Freq** | **Percent** | **Freq** | **Percent** |
| 0 | 81 | 22.5 | 161 | 18.38 | 0 | 101 | 28.06 | 227 | 25.91 |
| 1 | 168 | 46.67 | 251 | 28.65 | 1 | 154 | 42.78 | 289 | 32.99 |
| 2 | 64 | 17.78 | 222 | 25.34 | 2 | 61 | 16.94 | 185 | 21.12 |
| 3 | 31 | 8.61 | 146 | 16.67 | 3 | 30 | 8.33 | 81 | 9.25 |
| 4 | 16 | 4.44 | 87 | 9.93 | 4 | 14 | 3.89 | 84 | 9.59 |
| Total | 360 | 100 | 867 | 98.97 | Total | 360 | 100 | 866 | 98.86 |
| Turning in bed | **Freq** | **Percent** | **Freq** | **Percent** | **Tremor** | **Freq** | **Percent** | **Freq** | **Percent** |
| 0 | 142 | 39.44 | 277 | 31.62 | 0 | 32 | 8.89 | 189 | 21.58 |
| 1 | 129 | 35.83 | 378 | 43.15 | 1 | 94 | 26.11 | 360 | 41.1 |
| 2 | 43 | 11.94 | 111 | 12.67 | 2 | 123 | 34.11 | 212 | 24.2 |
| 3 | 33 | 9.17 | 55 | 6.28 | 3 | 87 | 24.17 | 72 | 8.22 |
| 4 | 13 | 3.61 | 50 | 5.71 | 4 | 24 | 6.67 | 36 | 4.11 |
| Total | 360 | 100 | 871 | 99.43 | Total | 360 | 100 | 869 | 99.2 |
| Getting out of bed | **Freq** | **Percent** | **Freq** | **Percent** | **Walking and balance** | **Freq** | **Percent** | **Freq** | **Percent** |
| 0 | 125 | 34.72 | 180 | 20.55 | 0 | 89 | 24.72 | 184 | 21 |
| 1 | 126 | 35 | 317 | 36.19 | 1 | 155 | 43.06 | 336 | 38.36 |
| 2 | 61 | 16.94 | 199 | 22.72 | 2 | 60 | 16.67 | 105 | 11.99 |
| 3 | 32 | 8.89 | 104 | 11.87 | 3 | 33 | 9.17 | 172 | 19.63 |
| 4 | 16 | 4.44 | 70 | 7.99 | 4 | 23 | 6.39 | 74 | 8.45 |
| Total | 360 | 100 | 870 | 99.32 | Total | 360 | 100 | 871 | 99.43 |
| Freezing | **Freq** | **Percent** | **Freq** | **Percent** |  | | | | |
| 0 | 155 | 43.06 | 453 | 51.71 |  |  |  |  |  |
| 1 | 105 | 29.17 | 182 | 20.78 |  |  |  |  |  |
| 2 | 50 | 13.89 | 89 | 10.16 |  |  |  |  |  |
| 3 | 35 | 9.72 | 90 | 10.27 |  |  |  |  |  |
| 4 | 15 | 4.17 | 56 | 6.39 |  |  |  |  |  |
| Total | 360 | 100 | 870 | 99.32 |  |  |  |  |  |

*Part 3*

|  | Kazakh | | English | |  | Kazakh | | English | |
| --- | --- | --- | --- | --- | --- | --- | --- | --- | --- |
| Speech | **Freq** | **Percent** | **Freq** | **Percent** | **Facial expression** | **Freq** | **Percent** | **Freq** | **Percent** |
| 0 | 166 | 46.11 | 189 | 21.58 | 0 | 92 | 25.56 | 96 | 10.96 |
| 1 | 122 | 33.89 | 379 | 43.26 | 1 | 151 | 41.94 | 300 | 34.25 |
| 2 | 55 | 15.28 | 213 | 24.32 | 2 | 78 | 21.67 | 361 | 41.21 |
| 3 | 12 | 3.33 | 69 | 7.88 | 3 | 33 | 9.17 | 89 | 10.16 |
| 4 | 5 | 1.39 | 22 | 2.51 | 4 | 6 | 1.67 | 26 | 2.97 |
| Total | 360 | 100 | 872 | 99.54 | Total | 360 | 100 | 872 | 99.54 |
| Rigidity (Neck) | **Freq** | **Percent** | **Freq** | **Percent** | **Rigidity (RUE)** | **Freq** | **Percent** | **Freq** | **Percent** |
| 0 | 131 | 36.39 | 260 | 29.68 | 0 | 119 | 33.06 | 176 | 20.09 |
| 1 | 122 | 33.89 | 247 | 28.2 | 1 | 104 | 28.89 | 282 | 32.19 |
| 2 | 74 | 20.56 | 274 | 31.28 | 2 | 88 | 24.44 | 342 | 39.04 |
| 3 | 28 | 7.78 | 73 | 8.33 | 3 | 45 | 12.5 | 69 | 7.88 |
| 4 | 5 | 1.39 | 16 | 1.83 | 4 | 4 | 1.11 | 6 | 0.68 |
| Total | 360 | 100 | 870 | 99.32 | Total | 360 | 100 | 875 | 99.89 |
| Rigidity (LUE) | **Freq** | **Percent** | **Freq** | **Percent** | **Rigidity (RLE)** | **Freq** | **Percent** | **Freq** | **Percent** |
| 0 | 130 | 36.11 | 205 | 23.4 | 0 | 161 | 44.72 | 272 | 31.05 |
| 1 | 113 | 31.39 | 268 | 30.59 | 1 | 117 | 32.5 | 248 | 28.31 |
| 2 | 84 | 23.33 | 317 | 36.19 | 2 | 58 | 16.11 | 275 | 31.39 |
| 3 | 25 | 6.94 | 77 | 8.79 | 3 | 20 | 5.56 | 67 | 7.65 |
| 4 | 8 | 2.22 | 7 | 0.8 | 4 | 4 | 1.11 | 10 | 1.14 |
| Total | 360 | 100 | 874 | 99.77 | Total | 360 | 100 | 872 | 99.54 |
| Rigidity (LLE) | **Freq** | **Percent** | **Freq** | **Percent** | **Finger tapping (R)** | **Freq** | **Percent** | **Freq** | **Percent** |
| 0 | 172 | 47.78 | 286 | 32.65 | 0 | 101 | 28.06 | 122 | 13.93 |
| 1 | 108 | 30 | 227 | 25.91 | 1 | 126 | 35 | 342 | 39.04 |
| 2 | 58 | 16.11 | 275 | 31.39 | 2 | 70 | 19.44 | 252 | 28.77 |
| 3 | 20 | 5.56 | 75 | 8.56 | 3 | 55 | 15.28 | 144 | 16.44 |
| 4 | 2 | 0.56 | 11 | 1.26 | 4 | 8 | 2.22 | 15 | 1.71 |
| Total | 360 | 100 | 874 | 99.77 | Total | 360 | 100 | 875 | 99.89 |
| Finger tapping (L) | **Freq** | **Percent** | **Freq** | **Percent** | **Hand movement (R)** | **Freq** | **Percent** | **Freq** | **Percent** |
| 0 | 125 | 34.72 | 108 | 12.33 | 0 | 123 | 34.17 | 187 | 21.35 |
| 1 | 117 | 32.5 | 298 | 34.02 | 1 | 125 | 34.72 | 346 | 39.5 |
| 2 | 79 | 21.94 | 265 | 30.25 | 2 | 61 | 16.94 | 231 | 26.37 |
| 3 | 32 | 8.89 | 181 | 20.66 | 3 | 44 | 12.22 | 98 | 11.19 |
| 4 | 7 | 1.94 | 22 | 2.51 | 4 | 7 | 1.94 | 12 | 1.37 |
| Total | 360 | 100 | 874 | 99.77 | Total | 360 | 100 | 874 | 99.77 |
| Hand movements (L) | **Freq** | **Percent** | **Freq** | **Percent** | **Pronation-supination movements (R)** | **Freq** | **Percent** | **Freq** | **Percent** |
| 0 | 150 | 41.67 | 164 | 18.72 | 0 | 130 | 36.11 | 199 | 22.72 |
| 1 | 123 | 34.17 | 311 | 35.5 | 1 | 114 | 31.67 | 335 | 38.24 |
| 2 | 59 | 16.39 | 250 | 28.54 | 2 | 65 | 18.06 | 216 | 24.66 |
| 3 | 21 | 5.83 | 125 | 14.27 | 3 | 44 | 12.22 | 107 | 12.21 |
| 4 | 7 | 1.94 | 25 | 2.85 | 4 | 7 | 1.94 | 17 | 1.94 |
| Total | 360 | 100 | 875 | 99.89 | Total | 360 | 100 | 874 | 99.77 |
| Pronation-supination movements (L) | **Freq** | **Percent** | **Freq** | **Percent** | **Toe tapping (R)** | **Freq** | **Percent** | **Freq** | **Percent** |
| 0 | 149 | 41.39 | 162 | 18.49 | 0 | 152 | 42.22 | 168 | 19.18 |
| 1 | 120 | 33.33 | 297 | 33.9 | 1 | 102 | 28.33 | 323 | 36.87 |
| 2 | 56 | 15.56 | 235 | 26.83 | 2 | 61 | 16.94 | 228 | 26.03 |
| 3 | 28 | 7.78 | 150 | 17.12 | 3 | 37 | 10.28 | 129 | 14.73 |
| 4 | 7 | 1.94 | 29 | 3.31 | 4 | 8 | 2.22 | 27 | 3.08 |
| Total | 360 | 100 | 873 | 99.66 | Total | 360 | 100 | 875 | 99.89 |
| Toe tapping (L) | **Freq** | **Percent** | **Freq** | **Percent** | **Leg agility (R)** | **Freq** | **Percent** | **Freq** | **Percent** |
| 0 | 155 | 43.06 | 154 | 17.58 | 0 | 168 | 46.67 | 250 | 28.54 |
| 1 | 112 | 31.11 | 251 | 28.65 | 1 | 96 | 26.67 | 329 | 37.56 |
| 2 | 60 | 16.67 | 268 | 30.59 | 2 | 52 | 14.44 | 190 | 21.69 |
| 3 | 23 | 6.39 | 154 | 17.58 | 3 | 37 | 10.28 | 86 | 9.82 |
| 4 | 10 | 2.78 | 46 | 5.25 | 4 | 7 | 1.94 | 18 | 2.05 |
| Total | 360 | 100 | 873 | 99.66 | Total | 360 | 100 | 873 | 99.66 |
| Leg agility (L) | **Freq** | **Percent** | **Freq** | **Percent** | **Arising from chair** | **Freq** | **Percent** | **Freq** | **Percent** |
| 0 | 173 | 48.06 | 216 | 24.66 | 0 | 201 | 55.83 | 422 | 48.17 |
| 1 | 96 | 26.67 | 298 | 34.02 | 1 | 82 | 22.78 | 245 | 27.97 |
| 2 | 61 | 16.94 | 213 | 24.32 | 2 | 40 | 11.11 | 78 | 8.9 |
| 3 | 21 | 5.83 | 106 | 12.1 | 3 | 22 | 6.11 | 71 | 8.11 |
| 4 | 9 | 2.5 | 38 | 4.34 | 4 | 15 | 4.17 | 55 | 6.28 |
| Total | 360 | 100 | 871 | 99.43 | Total | 360 | 100 | 871 | 99.43 |
| Gait | **Freq** | **Percent** | **Freq** | **Percent** | **Freezing of gait** | **Freq** | **Percent** | **Freq** | **Percent** |
| 0 | 65 | 18.06 | 202 | 23.06 | 0 | 210 | 58.33 | 655 | 74.77 |
| 1 | 174 | 48.33 | 351 | 40.07 | 1 | 82 | 22.78 | 95 | 10.84 |
| 2 | 67 | 18.61 | 167 | 19.06 | 2 | 34 | 9.44 | 60 | 6.85 |
| 3 | 37 | 10.28 | 97 | 11.07 | 3 | 22 | 6.11 | 26 | 2.97 |
| 4 | 17 | 4.72 | 55 | 6.28 | 4 | 12 | 3.33 | 38 | 4.34 |
| Total | 360 | 100 | 872 | 99.54 | Total | 360 | 100 | 874 | 99.77 |
| Postural stability | **Freq** | **Percent** | **Freq** | **Percent** | **Posture** | **Freq** | **Percent** | **Freq** | **Percent** |
| 0 | 139 | 38.61 | 422 | 48.17 | 0 | 72 | 20 | 173 | 19.75 |
| 1 | 107 | 29.72 | 157 | 17.92 | 1 | 181 | 50.28 | 337 | 38.47 |
| 2 | 58 | 16.11 | 60 | 6.85 | 2 | 74 | 20.56 | 206 | 23.52 |
| 3 | 39 | 10.83 | 149 | 17.01 | 3 | 25 | 6.94 | 125 | 14.27 |
| 4 | 17 | 4.72 | 86 | 9.82 | 4 | 8 | 2.22 | 33 | 3.77 |
| Total | 360 | 100 | 874 | 99.77 | Total | 360 | 100 | 874 | 99.77 |
| Global spontaneity of movements | **Freq** | **Percent** | **Freq** | **Percent** | **Postural tremor (R)** | **Freq** | **Percent** | **Freq** | **Percent** |
| 0 | 101 | 28.06 | 108 | 12.33 | 0 | 140 | 38.89 | 544 | 62.1 |
| 1 | 151 | 41.94 | 278 | 31.74 | 1 | 126 | 35 | 262 | 29.91 |
| 2 | 58 | 16.11 | 279 | 31.85 | 2 | 69 | 19.17 | 43 | 4.91 |
| 3 | 33 | 9.17 | 184 | 21 | 3 | 17 | 4.72 | 23 | 2.63 |
| 4 | 17 | 4.72 | 27 | 3.08 | 4 | 8 | 2.22 | 1 | 0.11 |
| Total | 360 | 100 | 876 | 100 | Total | 360 | 100 | 873 | 99.66 |
| Postural tremor (L) | **Freq** | **Percent** | **Freq** | **Percent** | **Kinetic tremor (R)** | **Freq** | **Percent** | **Freq** | **Percent** |
| 0 | 172 | 47.78 | 518 | 59.13 | 0 | 149 | 41.39 | 546 | 62.33 |
| 1 | 122 | 33.89 | 276 | 31.51 | 1 | 145 | 40.28 | 265 | 30.25 |
| 2 | 45 | 12.5 | 49 | 5.59 | 2 | 44 | 12.22 | 46 | 5.25 |
| 3 | 15 | 4.17 | 29 | 3.31 | 3 | 14 | 3.89 | 13 | 1.48 |
| 4 | 6 | 1.67 | 1 | 0.11 | 4 | 8 | 2.22 | 2 | 0.23 |
| Total | 360 | 100 | 873 | 99.66 | Total | 360 | 100 | 872 | 99.54 |
| Kinetic tremor (L) | **Freq** | **Percent** | **Freq** | **Percent** | **Rest tremor amplitude (RUE)** | **Freq** | **Percent** | **Freq** | **Percent** |
| 0 | 166 | 46.11 | 493 | 56.28 | 0 | 152 | 42.22 | 586 | 66.89 |
| 1 | 139 | 38.61 | 293 | 33.45 | 1 | 84 | 23.33 | 112 | 12.79 |
| 2 | 39 | 10.83 | 72 | 8.22 | 2 | 84 | 23.33 | 121 | 13.81 |
| 3 | 5 | 1.39 | 14 | 1.6 | 3 | 26 | 7.22 | 53 | 6.05 |
| 4 | 11 | 3.06 | 0 | 0 | 4 | 14 | 3.89 | 3 | 0.34 |
| Total | 360 | 100 | 872 | 99.54 | Total | 360 | 100 | 875 | 99.89 |
| Rest tremor amplitude (LUE) | **Freq** | **Percent** | **Freq** | **Percent** | **Rest tremor amplitude (RLE)** | **Freq** | **Percent** | **Freq** | **Percent** |
| 0 | 200 | 55.56 | 603 | 68.84 | 0 | 254 | 70.56 | 777 | 88.7 |
| 1 | 68 | 18.89 | 120 | 13.7 | 1 | 52 | 14.44 | 52 | 5.94 |
| 2 | 68 | 18.89 | 99 | 11.3 | 2 | 39 | 10.83 | 35 | 4 |
| 3 | 17 | 4.72 | 45 | 5.14 | 3 | 11 | 3.06 | 9 | 1.03 |
| 4 | 7 | 1.94 | 5 | 0.57 | 4 | 4 | 1.11 | 0 | 0 |
| Total | 360 | 100 | 872 | 99.54 | Total | 360 | 100 | 873 | 99.66 |
| Rest tremor amplitude (LLE) | **Freq** | **Percent** | **Freq** | **Percent** | **Rest tremor amplitude (Lip/Jaw)** | **Freq** | **Percent** | **Freq** | **Percent** |
| 0 | 274 | 76.11 | 795 | 90.75 | 0 | 263 | 73.06 | 780 | 89.04 |
| 1 | 46 | 12.78 | 46 | 5.25 | 1 | 64 | 17.78 | 63 | 7.19 |
| 2 | 28 | 7.78 | 20 | 2.28 | 2 | 21 | 5.83 | 18 | 2.05 |
| 3 | 6 | 1.67 | 12 | 1.37 | 3 | 6 | 1.67 | 13 | 1.48 |
| 4 | 6 | 1.67 | 0 | 0 | 4 | 6 | 1.67 | 1 | 0.11 |
| Total | 360 | 100 | 873 | 99.66 | Total | 360 | 100 | 875 | 99.89 |
| Constancy of rest | **Freq** | **Percent** | **Freq** | **Percent** |  | | | | |
| 0 | 78 | 21.67 | 409 | 46.69 |  |  |  |  |  |
| 1 | 52 | 14.44 | 214 | 24.43 |  |  |  |  |  |
| 2 | 71 | 19.72 | 91 | 10.39 |  |  |  |  |  |
| 3 | 70 | 19.44 | 85 | 9.7 |  |  |  |  |  |
| 4 | 89 | 24.72 | 67 | 7.65 |  |  |  |  |  |
| Total | 360 | 100 | 866 | 98.86 |  |  |  |  |  |

*Part 4*

|  | Kazakh | | English | |  | Kazakh | | English | |
| --- | --- | --- | --- | --- | --- | --- | --- | --- | --- |
| Time spent with dyskinesias | **Freq** | **Percent** | **Freq** | **Percent** | **Functional impact of dyskinesias** | **Freq** | **Percent** | **Freq** | **Percent** |
| 0 | 178 | 49.44 | 563 | 64.27 | 0 | 195 | 54.17 | 695 | 79.94 |
| 1 | 78 | 21.67 | 173 | 19.75 | 1 | 78 | 21.67 | 90 | 10.27 |
| 2 | 54 | 15 | 87 | 9.93 | 2 | 55 | 15.28 | 29 | 3.31 |
| 3 | 29 | 8.06 | 27 | 3.08 | 3 | 22 | 6.11 | 46 | 5.25 |
| 4 | 21 | 5.83 | 17 | 1.94 | 4 | 10 | 2.78 | 5 | 0.57 |
| Total | 360 | 100 | 867 | 98.97 | Total | 360 | 100 | 865 | 98.74 |
| Time spent in OFF state | **Freq** | **Percent** | **Freq** | **Percent** | **Functional impact of fluctuations** | **Freq** | **Percent** | **Freq** | **Percent** |
| 0 | 112 | 31.11 | 383 | 43.72 | 0 | 123 | 34.17 | 433 | 49.43 |
| 1 | 113 | 31.39 | 341 | 38.93 | 1 | 108 | 30 | 165 | 18.84 |
| 2 | 111 | 30.83 | 106 | 12.1 | 2 | 65 | 18.06 | 81 | 9.25 |
| 3 | 19 | 5.28 | 22 | 2.51 | 3 | 52 | 14.44 | 119 | 13.58 |
| 4 | 5 | 1.39 | 14 | 1.6 | 4 | 12 | 3.33 | 63 | 7.19 |
| Total | 360 | 100 | 866 | 98.86 | Total | 360 | 100 | 861 | 98.29 |
| Complexity of motor fluctuations | **Freq** | **Percent** | **Freq** | **Percent** | **Painful OFF-state dystonia** | **Freq** | **Percent** | **Freq** | **Percent** |
| 0 | 114 | 31.67 | 404 | 46.12 | 0 | 134 | 37.22 | 680 | 77.63 |
| 1 | 151 | 41.94 | 291 | 33.22 | 1 | 121 | 33.61 | 114 | 13.01 |
| 2 | 62 | 17.22 | 69 | 7.88 | 2 | 74 | 20.56 | 45 | 5.14 |
| 3 | 25 | 6.94 | 50 | 5.71 | 3 | 24 | 6.67 | 13 | 1.48 |
| 4 | 8 | 2.22 | 46 | 5.25 | 4 | 7 | 1.94 | 15 | 1.71 |
| Total | 360 | 100 | 860 | 98.17 | Total | 360 | 100 | 867 | 98.97 |

**Table 2**

*Exploratory Factor Analysis (EFA)*

*Part 1:* Non-Motor Aspects of Experiences of Daily Living

|  | | Kazakh | English |
| --- | --- | --- | --- |
| Factor 1 | Cognitive impairment | 0.471 |  |
|  | Hallucinations and psychosis | 0.510 |  |
|  | Depressed mood | 0.563 | 0.816 |
|  | Anxious mood | 0.787 | 0.667 |
|  | Apathy | 0.781 | 0.550 |
|  | Features of DDS | 0.489 |  |
|  | Fatigue | 0.469 |  |
| Factor 2 | Cognitive impairment | 0.564 | 0.550 |
|  | Hallucinations and psychosis | 0.523 | 0.579 |
|  | Depressed mood | 0.411 |  |
|  | Features of DDS | 0.632 |  |
|  | Sleep problems | 0.592 |  |
|  | Daytime sleepiness | 0.553 | 0.534 |
|  | Pain and other sensations | 0.405 | 0.441 |
|  | Urinary problems | 0.747 | 0.597 |
|  | Constipation problems | 0.447 | 0.458 |
|  | Lightheadedness on standing | 0.582 | 0.458 |
|  | Fatigue | 0.573 | 0.482 |

*(CFI = 0.974, RMSEA = 0.07)*

*Part 2:* Motor Aspects of Experiences of Daily Living

|  | | Kazakh | English |
| --- | --- | --- | --- |
| Factor 1 | Speech | 0.664 | 0.774 |
|  | Saliva and drooling | 0.636 | 0.438 |
|  | Chewing and swallowing | 0.802 | 0.598 |
|  | Eating tasks | 0.517 | 0.408 |
|  | Dressing | 0.683 |  |
|  | Hygiene | 0.490 |  |
|  | Turning in bed | 0.640 |  |
|  | Tremor | 0.758 |  |
|  | Handwriting |  | 0.490 |
|  | Doing hobbies and other activities |  | 0.481 |
| Factor 2 | Speech | 0.466 |  |
|  | Saliva ang drooling | 0.501 |  |
|  | Eating tasks | 0.765 |  |
|  | Dressing | 0.531 | 0.612 |
|  | Hygiene | 0.781 | 0.616 |
|  | Getting out of bed | 0.450 | 0.719 |
|  | Walking and balance | 0.446 | 0.817 |
|  | Freezing | 0.435 | 0.776 |
|  | Doing hobbies and other activities |  | 0.418 |
|  | Turning in bed |  | 0.635 |
| Factor 3 | Chewing and swallowing | 0.405 |  |
|  | Handwriting | 0.796 |  |
|  | Doing hobbies and other activities | 0.812 |  |
|  | Turning in bed | 0.588 |  |
|  | Getting out of bed | 0.539 |  |
|  | Freezing | 0.439 |  |
|  | Eating tasks |  | 0.648 |
|  | Dressing |  | 0.574 |
|  | Hygiene |  | 0.505 |
|  | Tremor |  | 0.412 |

*(CFI = 0.996, RMSEA = 0.061)*

*Part 3:* Motor Examination

|  | | Kazakh | English |
| --- | --- | --- | --- |
| Factor 1 | Facial expression | 0.870 | 0.577 |
|  | Rigidity (LUE) | 0.840 |  |
|  | Rigidity (RLE) | 0.413 |  |
|  | Rigidity (LLE) | 0.429 |  |
|  | Finger tapping (L) | 0.836 |  |
|  | Hand movements (L) | 0.893 |  |
|  | Pronation-supination movements (L) | 0.826 |  |
|  | Toe tapping (L) | 0.891 |  |
|  | Leg agility (R) | 0.619 | 0.424 |
|  | Leg agility (L) | 0.567 |  |
|  | Gait | 0.424 | 0.878 |
|  | Postural stability | 0.458 | 0.802 |
|  | Global spontaneity of movement | 0.578 | 0.668 |
|  | Postural tremor (L) | 0.578 |  |
|  | Kinetic tremor (L) | 0.576 |  |
|  | Rest tremor amplitude (RLE) | 0.549 |  |
|  | Rest tremor amplitude (LLE) | 0.519 |  |
|  | Rest tremor (Lip/Jaw) | 0.530 |  |
|  | Constancy of rest | 0.577 |  |
|  | Speech |  | 0.610 |
|  | Arising from chair |  | 0.815 |
|  | Freezing of gait |  | 0.826 |
|  | Posture |  | 0.690 |
| Factor 2 | Rigidity (Neck) | 0.789 |  |
|  | Rigidity (RUE) | 0.775 |  |
|  | Rigidity (RLE) | 0.811 |  |
|  | Rigidity (LLE) | 0.794 |  |
|  | Finger tapping (R) | 0.807 |  |
|  | Leg agility (L) | 0.769 |  |
|  | Arising from chair | 0.516 |  |
|  | Gait | 0.717 |  |
|  | Global spontaneity of movement | 0.479 |  |
|  | Postural tremor (R) | 0.412 | 0.406 |
|  | Postural tremor (L) | 0.505 |  |
|  | Kinetic tremor (L) | 0.503 |  |
|  | Rest tremor amplitude (RUE) | 0.459 | 0.767 |
|  | Rest tremor amplitude (RLE) | 0.443 | 0.728 |
|  | Rest tremor amplitude (LLE) | 0.449 | 0.698 |
|  | Rest tremor amplitude (Lip/Jaw) | 0.509 | 0.582 |
|  | Constancy of rest | 0.568 | 0.941 |
|  | Rest tremor amplitude (LUE) |  | 0.753 |
| Factor 3 | Speech | 0.852 |  |
|  | Hand movements (R) | 0.871 | 0.720 |
|  | Pronation-supination movements (R) | 0.850 | 0.687 |
|  | Toe tapping (R) | 0.817 | 0.412 |
|  | Freezing of gait | 0.404 |  |
|  | Postural stability | 0.459 |  |
|  | Global spontaneity of movement | 0.430 |  |
|  | Postural tremor (L) | 0.463 |  |
|  | Kinetic tremor (L) | 0.472 |  |
|  | Rest tremor amplitude (RUE) | 0.501 |  |
|  | Rest tremor amplitude (RLE) | 0.451 |  |
|  | Finger tapping (R) |  | 0.698 |
|  | Leg agility (R) |  | 0.405 |
| Factor 4 | Arising from chair | 0.526 |  |
|  | Freezing of gait | 1.029 |  |
|  | Postural stability | 0.443 |  |
|  | Posture | 0.447 |  |
|  | Postural tremor (R) | 0.635 | 0.654 |
|  | Kinetic tremor (R) | 0.656 | 0.849 |
|  | Rest tremor amplitude (LLE) | 0.420 |  |
|  | Postural tremor (L) |  | 0.692 |
|  | Kinetic tremor (L) |  | 0.845 |
| Factor 5 | Postural tremor (R) | 0.423 |  |
|  | Kinetic tremor (R) | 0.425 |  |
|  | Rest tremor amplitude (RUE) | 0.461 |  |
|  | Rest tremor amplitude (LUE) | 1.279 |  |
|  | Rest tremor amplitude (LLE) | 0.458 |  |
|  | Rigidity (Neck) |  | 0.652 |
|  | Rigidity (RUE) |  | 0.717 |
|  | Rigidity (LUE) |  | 0.711 |
|  | Rigidity (RLE) |  | 0.852 |
|  | Rigidity (LLE) |  | 0.870 |
| Factor 6 | Finger tapping (L) |  | 0.658 |
|  | Hand movements (L) |  | 0.702 |
|  | Pronation-supination movements (L) |  | 0.636 |
|  | Toe tapping (L) |  | 0.436 |
| Factor 7 | Toe tapping (R) |  | 0.667 |
|  | Toe tapping (L) |  | 0.682 |
|  | Leg agility (R) |  | 0.659 |
|  | Leg agility (L) |  | 0.672 |

*(CFI = 0.993, RMSEA = 0.053)*

*Part 4:* Motor Complications

|  |  | Kazakh | English |
| --- | --- | --- | --- |
| Factor 1 | Time spent with dyskinesias | 0.803 |  |
|  | Functional impact of dyskinesias | 0.779 |  |
|  | Time spent in the OFF state | 0.670 | 0.866 |
|  | Functional impact of fluctuations | 0.597 | 0.846 |
|  | Complexity of motor fluctuations | 0.599 | 0.824 |
|  | Painful OFF-state dystonia | 0.631 | 0.501 |
| Factor 2 | Time spent with dyskinesias | 0.596 | 0.738 |
|  | Functional impact of dyskinesias | 0.628 | 0.914 |
|  | Time spent in the OFF state | 0.739 |  |
|  | Functional impact of fluctuations | 0.808 |  |
|  | Complexity of motor fluctuations | 0.793 |  |
|  | Painful OFF-state dystonia | 0.771 |  |

*(CFI = 1, RMSEA = 0)*

**A**

**
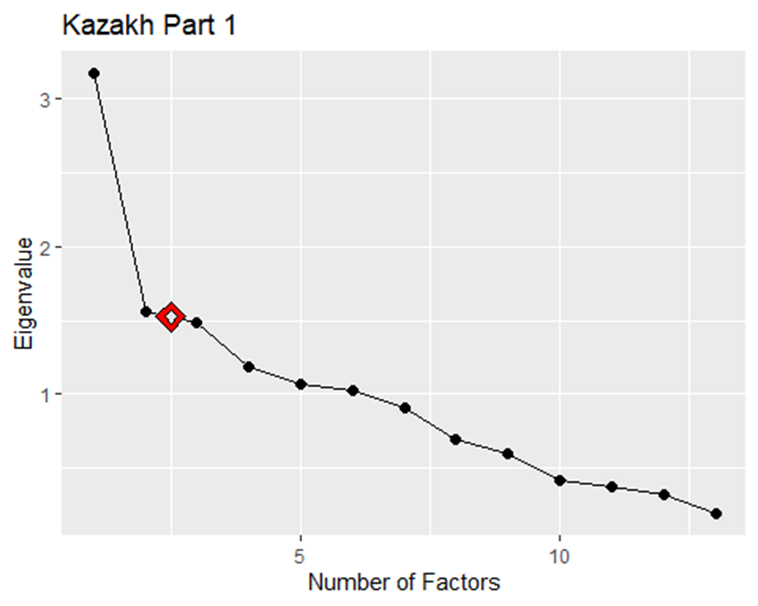
**

**B**

**
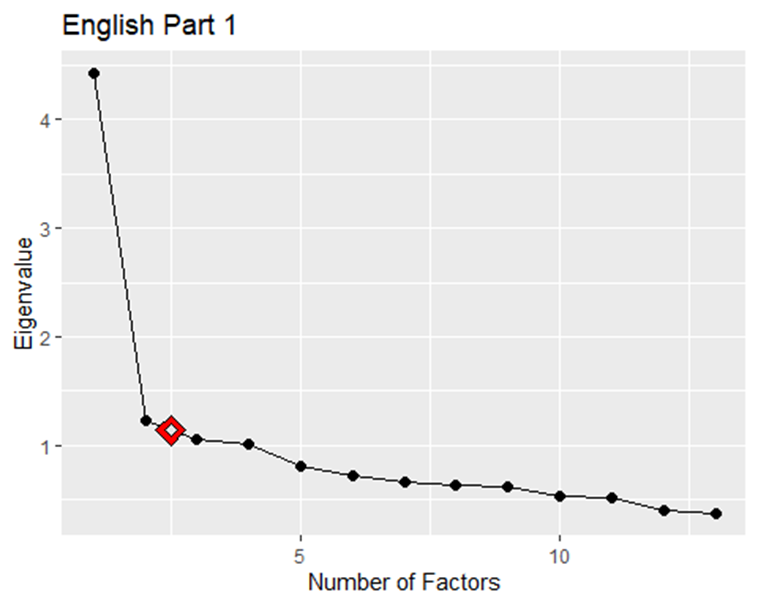
**

**Supplementary Figure 1.** The number of factors and their associated eigenvalues and percent variance of the results of the EFA for the for Non-Motor Aspects of Experiences of Daily Living Part 1. A) Kazakh version; B) English version

**EFA-** Exploratory Factor Analysis.

**A**

**
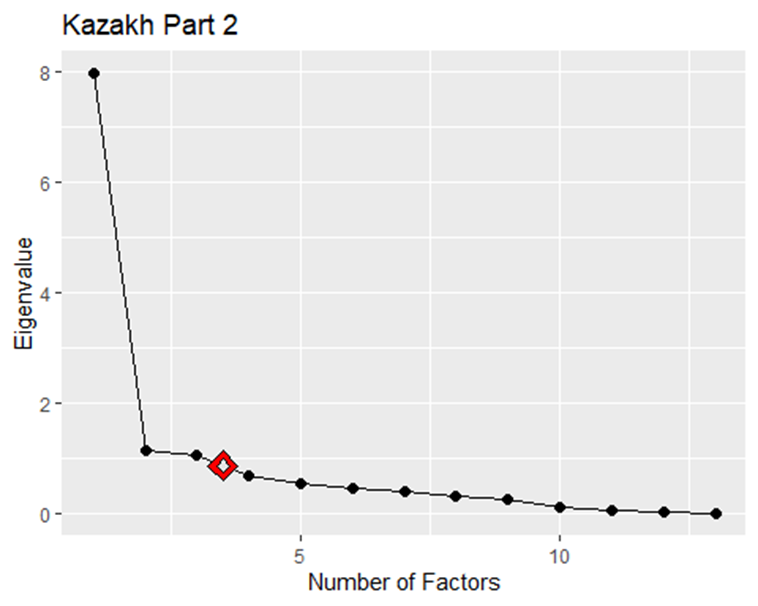
**

**B**

**
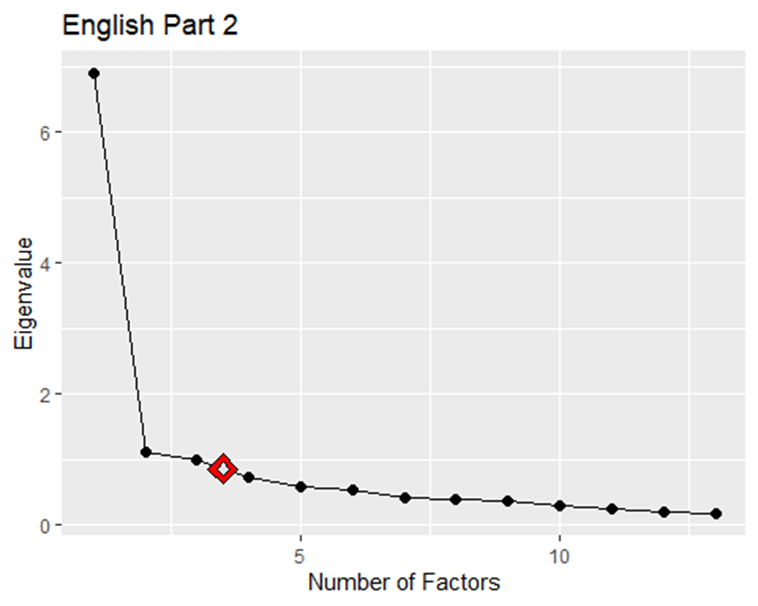
**

**Supplementary Figure 2.** The number of factors and their associated eigenvalues and percent variance of the results of the EFA for the for Non-Motor Aspects of Experiences of Daily Living Part 2. A) Kazakh version; B) English version

**A**

**
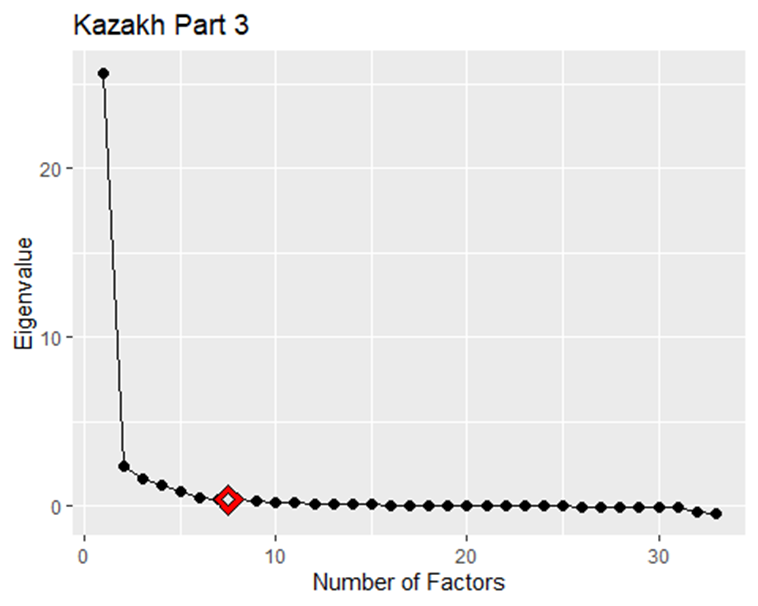
**

**B**

**
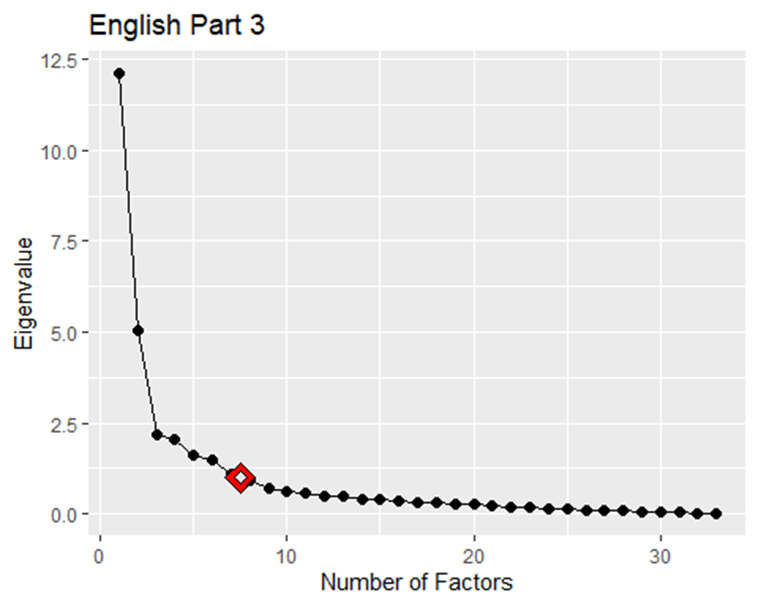
**

**Supplementary Figure 3.** The number of factors and their associated eigenvalues and percent variance of the results of the EFA for motor examination. A) Kazakh version; B) English version

**A**


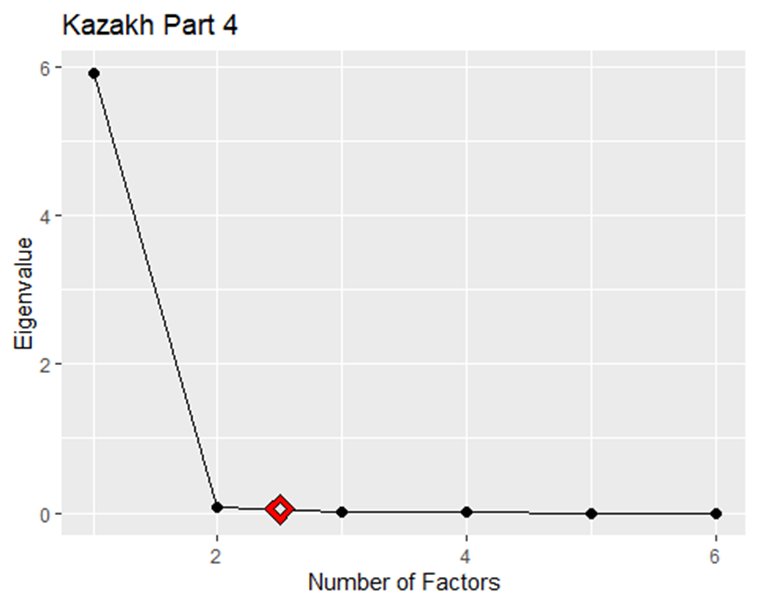


**B**

**
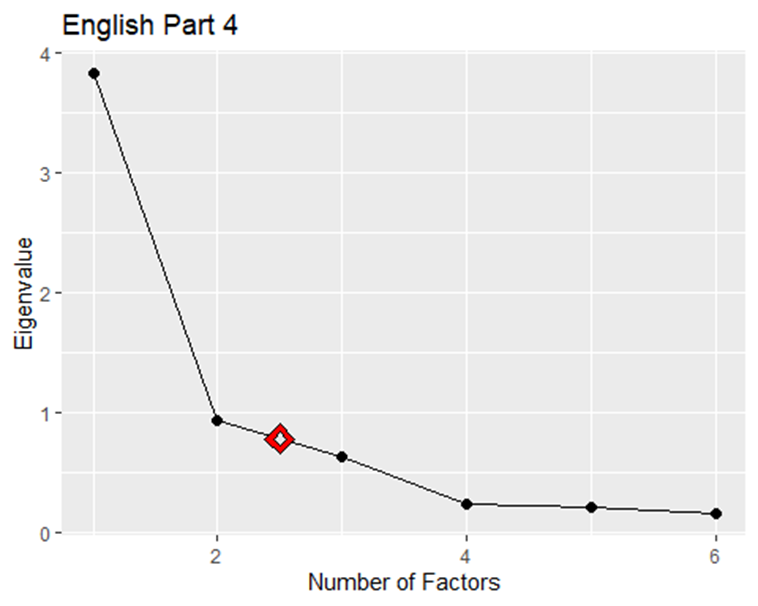
**

**Supplementary Figure 4.** The number of factors and their associated eigenvalues and percent variance of the results of the EFA for motor complications. A) Kazakh version; B) English version
